# Development and Application of Digital Maxillofacial Surgery System Based on Mixed Reality Technology

**DOI:** 10.3389/fsurg.2021.719985

**Published:** 2022-01-31

**Authors:** Rong Yang, Chenyao Li, Puxun Tu, Abdelrehem Ahmed, Tong Ji, Xiaojun Chen

**Affiliations:** ^1^Shanghai Key Laboratory of Stomatology/Shanghai Institute of Stomatology, Department of Oral and Maxillofacial Head and Neck Oncology, National Clinical Research Center for Oral Diseases, School of Medicine, The Ninth People's Hospital, Shanghai Jiao Tong University, Shanghai, China; ^2^School of Mechanical and Engineering, Shanghai Jiaotong University, Shanghai, China; ^3^Department of Craniomaxillofacial and Plastic Surgery, Faculty of Dentistry, Alexandria University, Alexandria, Egypt

**Keywords:** maxillofacial surgery, digital surgery, surgical navigation, mixed reality, three-dimensional visual output

## Abstract

**Objective:**

To realize the three-dimensional visual output of surgical navigation information by studying the cross-linking of mixed reality display devices and high-precision optical navigators.

**Methods:**

Applying quaternion-based point alignment algorithms to realize the positioning configuration of mixed reality display devices, high-precision optical navigators, real-time patient tracking and calibration technology; based on open source SDK and development tools, developing mixed reality surgery based on visual positioning and tracking system. In this study, four patients were selected for mixed reality-assisted tumor resection and reconstruction and re-examined 1 month after the operation. We reconstructed postoperative CT and use 3DMeshMetric to form the error distribution map, and completed the error analysis and quality control.

**Results:**

Realized the cross-linking of mixed reality display equipment and high-precision optical navigator, developed a digital maxillofacial surgery system based on mixed reality technology and successfully implemented mixed reality-assisted tumor resection and reconstruction in 4 cases.

**Conclusions:**

The maxillofacial digital surgery system based on mixed reality technology can superimpose and display three-dimensional navigation information in the surgeon's field of vision. Moreover, it solves the problem of visual conversion and space conversion of the existing navigation system. It improves the work efficiency of digitally assisted surgery, effectively reduces the surgeon's dependence on spatial experience and imagination, and protects important anatomical structures during surgery. It is a significant clinical application value and potential.

## Background

The combination of surgery and digital navigation technology is the international mainstream and frontier development trend in this field. In the oral and maxillofacial regions, the rigidity characteristics of the skull base and maxillofacial bones provide an anatomical basis for the clinical application of digital navigation technology (1). Existing digital technologies such as surgical planning, intraoperative real-time navigation, and others have significantly improved oral and maxillofacial surgery accuracy. They can avoid secondary injuries to the greatest extent on the premise of completing the established resection goals of the operation (2). The advantages of current digital navigation technology are summarized as follows: (1) Precisely locate the anatomical structures and pathological tissues of the operation area; (2) Perfect surgical planning and approach before surgery; (3) Precisely control the resection range to protect important anatomical structures; (4) Accurate Repair the morphology of maxillofacial defect (3).

Although the existing digital navigation system-assisted surgical system has matured day by day, there are still some problems. (1) There is no direct spatial relationship between the patient and the surgical planning and the output image of the navigation system. The surgeon needs to watch the tomographic image output by the surgical planning and the navigation system through the display screen. When using images to locate anatomical structures, the surgeon needs to imagine the actual relationship between the image and the patient's anatomy, which requires a solid anatomical foundation and robust spatial imagination ability, which raises the technical threshold (4). (2) The existing navigation system is single-point navigation. It means that it can see only a particular point of navigation information during the operation but not a full grasp of the surgical area (5). (3) The existing navigation system is a display screen output. During use, the surgeon needs to repeatedly switch the field of vision between the display screen and the operation area. In addition to affecting the operation process and reducing the operation efficiency, it also quickly causes the doctor's visual fatigue and affects the operation effect (6). The above problems limit the further promotion and development of the existing navigation system in oral and maxillofacial surgery.

With improved computing capabilities and image processing technology, it has become possible to introduce augmented reality and mixed reality technologies into the medical field. Mixed reality technology integrates the technical advantages of augmented reality and virtual reality, can present virtual model information in natural scenes, integrate virtual models and real locations, and create a better realistic experience through stage superimposition (7). Intraoperative navigation is one of the best application scenarios of mixed reality technology in the medical field. Mixed reality equipment can assist the navigation system in integrating the surgical planning three-dimensional model with the actual patient's anatomy during the operation and complete the continuous spatiotemporal registration of the patient's surgical area anatomy and the planned three-dimensional model. Mixed reality technology can extend the surgeon's field of vision, enhance the visual system, and obtain the internal structure of organs invisible to the naked eye. The accurate spatial information of the surgical site and surrounding critical anatomical structures are further obtained (8).

Therefore, the introduction of mixed reality technology into the navigation system of oral and maxillofacial surgery can realize three-dimensional visual navigation and better solve spatial imagination and graphic conversion of existing navigation systems. The following introduces the project team's cross-linking of mixed reality display equipment with high-precision optical navigators, developing a digital maxillofacial surgery system based on mixed reality technology, and successfully implemented mixed reality-assisted tumor resection and reconstruction in 4 cases.

## Methods

### MR Display Equipment and Patient Tracking Calibration Technology

The dynamic tracking of the optical positioner mainly realizes the change from real space to MR space. The process has two steps: the change from the real space to the space of the optical positioner and the change from the distance of the optical positioner to the MR display device. The reference frame fixed on the patient's head and the reflective ball on the MR display device's positioning frame can define the real space and the MR space. Using the optical positioner to track the reflective balls on the two positioning frames can directly obtain the change from the positioner to the MR space. Sum these two changes can get the transformation relationship from the real space to the MR space. Moreover, the MR equipment can scan the operating room environment through the depth camera for auxiliary calibration, further improving the accuracy of the tracking calibration.

### Development of Maxillofacial Digital Surgery System Based on Mixed Reality Technology

The hardware used in this system includes HoloLens, a commercial head-mounted mixed reality display device from Microsoft Corporation in the United States, Passive Polaris Spectra, an infrared positioning tracking device from NDI Corporation in Canada, and infrared-reflective balls and reference frames.The software architecture used in this system includes HoloLens application development kit Mixed Reality Toolkit, Unity3D game engine for middleware development, open-source toolkit IGSTK developed by the National Institute of Biomedical Imaging and Bioengineering, National Institutes of Health, cross-platform QT, and communication-based on TCP protocol. The software used in this system is a commercial development kit or an open-source kit.

### Preliminary Clinical Application of Maxillofacial Digital Surgery System Based on Mixed Reality Technology

A total of four patients who were treated at the Ninth People's Hospital of Shanghai Jiaotong University were selected. All patients had mandibular tumors that needed resection and reconstruction. We collected the patient's original CT data, completed the patient's preoperative three-dimensional reconstruction and surgical planning, and used the mixed reality technology of the maxillofacial digital surgery system to assist the surgery (Table 1).

**Table 1:** Information of the 4 patients and surgical results.

**Case**	**Gander**	**Age/years**	**Number of fibula bone segments**	**Maximum error/mm**	**Type of defect**	**Cause of defect**	**Benign or Malignant**
A	Female	37	2	5.46	Metal and body	Mandible tumor	Benign
B	Male	46	1	5.80	Metal and body	Mandible tumor	Benign
C	Male	45	2	6.08	Metal and body	Mandible tumor	Benign
D	Female	54	3	4.79	Bilateral	Mandible tumor	Malignant

The patients were re-examined 1 month after the operation, the postoperative CT data of the patient were collected, and the data were processed through the 3DMeshMetric to form the error distribution map, and completed the error analysis and quality control.

## Results

### MR Display Equipment and Patient Tracking Calibration Technology

The preoperative CT or MRI inspection data can be transformed into a virtual visual model through three-dimensional modeling. Select the anatomical landmarks accurately identified in the surgical site as the marker points. Calculate the coordinates of the marked points on the virtual model in the original coordinate system of the virtual model, denoted as {Mi}, which is the coordinates of the patient's accurate anatomical marked points in the actual model coordinate system. During the operation, the navigation system tracks the surgical probe. The anatomical landmarks preset as the marker points on the actual model are sequentially selected to obtain the position of the marker point in the coordinate system of the navigation device. Click the anatomical landmarks preset as marker points on the actual model in turn to obtain the position of the marker point in the coordinate system of the navigation device, and further calculate the coordinates of the marker point in the reference frame coordinate system of the actual model, denoted as {Ni}.

**Figure 1:**
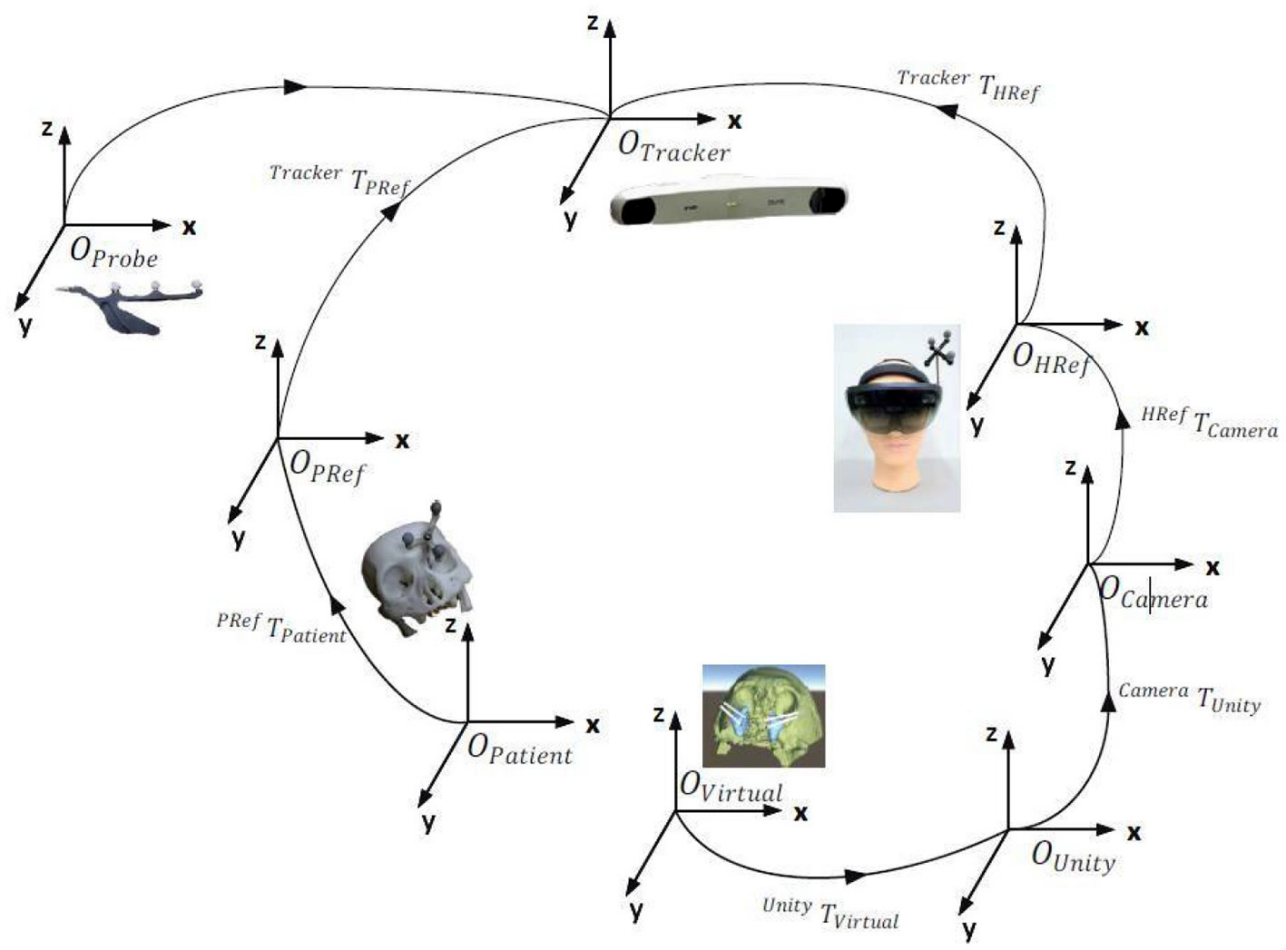
Tracking calibration of MR display equipment, navigation system, and actual patients.

Use the least-square method to find the optimal transformation matrix NTM to make the transformed coordinates NTM (Mi) to (Ni) closer to complete the tracking calibration of the MR display device and the patient. When performing point registration, as the number of marker points increases, the registration error will decrease. However, too many marker points in the actual operation will prolong the registration time and affect the efficiency of the process (9). Based on the research data, we set the number of marking points to six and control the point registration error within 1 mm, which achieves a balance of accuracy and efficiency (Figure 1).

### Development of Maxillofacial Digital Surgery System Based on Mixed Reality Technology

The surgical system software system includes MR image processing and selection module, registration module, control module, target area depth calculation module. The design interface of the software system is simple and straightforward. It can display the MR virtual three-dimensional model and CT/MRI cross-section conditions simultaneously, switch arbitrarily, and realize the distance measurement and real-time display of the target area and the position of the surgical instrument.

MR image processing and selection module: including 3D model rendering optimization algorithm, image segmentation selection algorithm and display, mainly used for MR image processing optimization and separate production; registration module: assists the virtual and actual matching of the MR 3D model and the patient's factual anatomical structure Accuracy, including the extraction and matching of virtual and real landmarks, and the identification of landmarks and positioning frames by the navigator; control module: the surgeon mainly operates and sets the entire navigation system through this module, including the hardware interface program of the navigator and MR display equipment; Target area depth calculation module: Calculate the depth of the surgical instrument and the target area, and optimize the navigation accuracy of the target area. The hardware system required for the prototype includes MR image display equipment, navigator, reference system (probe, positioning frame, reflector...). The MR image display device completes the real-time communication with the navigator through the data transmission interface based on the TCP/UDP transmission protocol.

**Figure 2:**
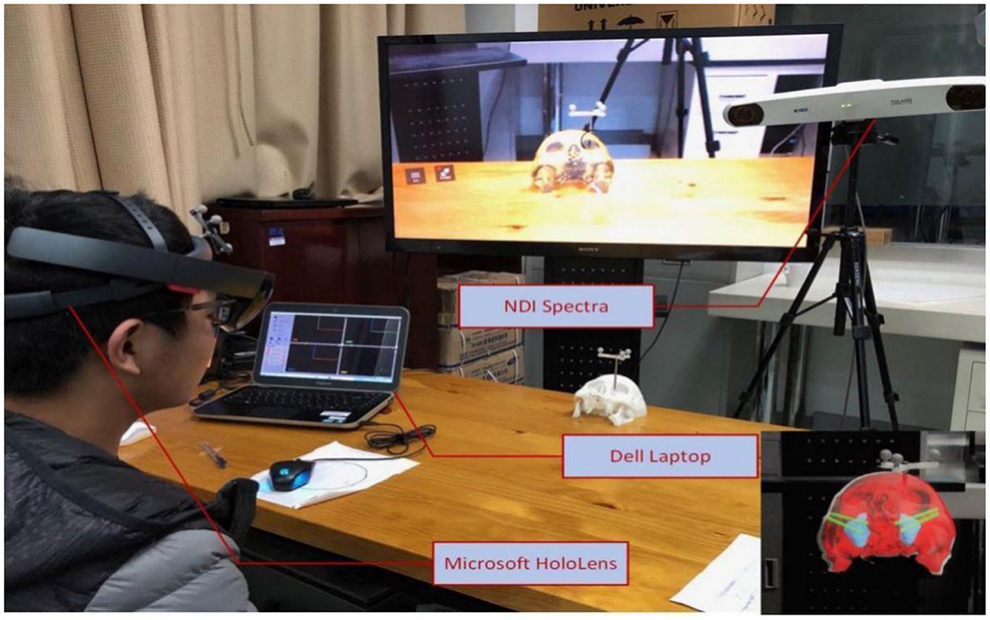
Construction of a prototype of a digital maxillofacial surgery system based on mixed reality technology.

The MR image display device is the key to the navigation system. This article uses the finished head-mounted wireless MR display device-HoloLens as the display device. The image rendering optimization, intraoperative registration, tracking calibration algorithm, and hardware real-time communication protocol involved are all designed and developed by this project team (Figure 2).

### Preliminary Clinical Application of Maxillofacial Digital Surgery System Based on Mixed Reality Technology

**Figure 3:**
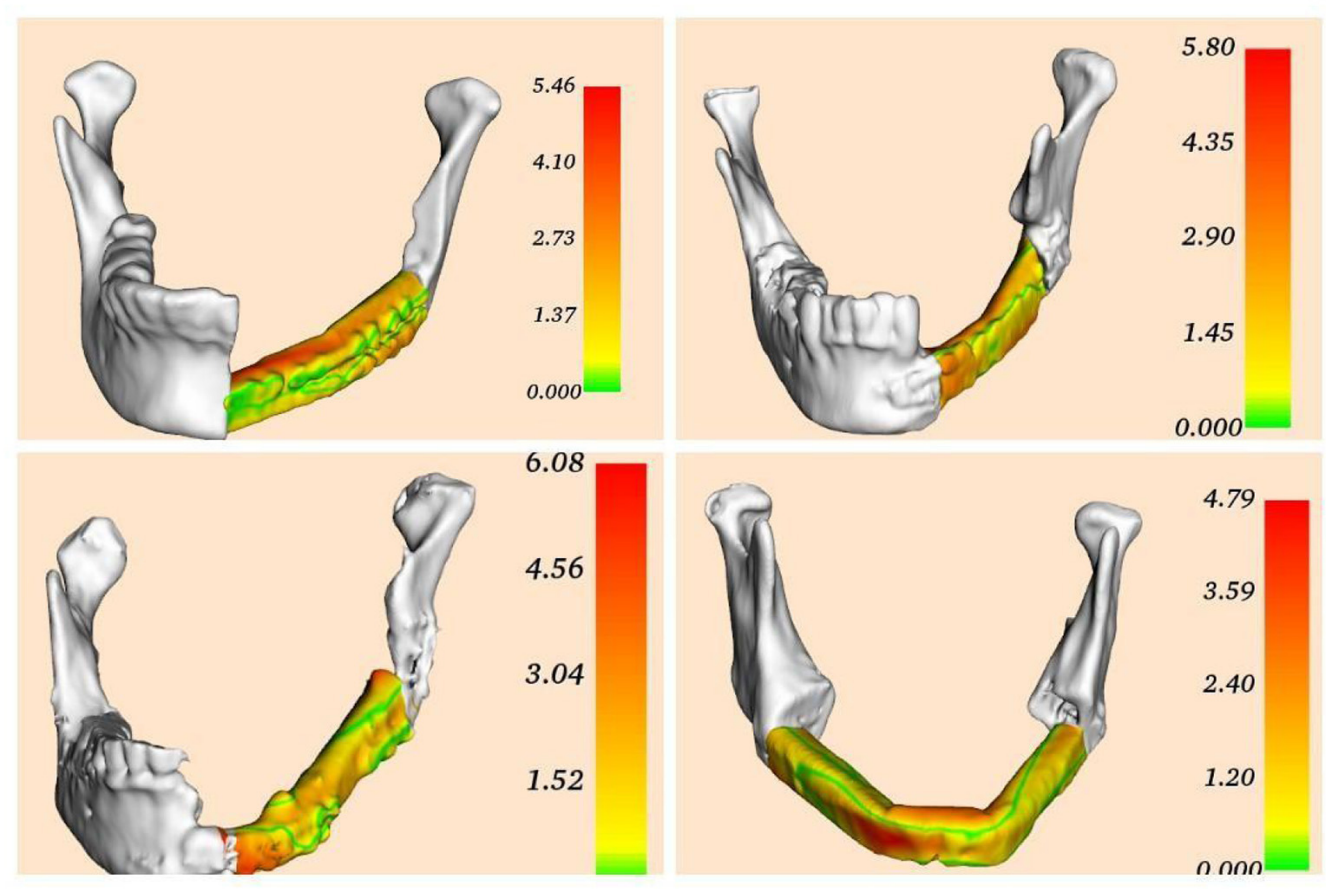
Error distribution diagram formed by point-to-point comparison between preoperative planning and model postoperative CT reconstruction model. The bar graph on the right represents the value of the error (unit: mm). The color in the figure corresponds to the point error.

The operation of the four patients went smoothly. During the operation, the surgeon could observe the operation plan and virtual three-dimensional model in the operation field in real time, and adjust it through voice and gesture operations. In all cases, the mandible and other anatomical structures were well displayed, and the information response of the reconstructed fibula was accurate.By comparing the CT and surgical planning models before and after the operation, and through the error analysis chart, the maximum error of all patients is not more than 6.08 mm, and the error in most areas is <4.79 mm. Besides, The errors in all cases are mainly concentrated in the chin, which may be caused by the difference between the surface of the chin and the shape of the fibula. The error of the selected case in this article meets the requirements, and the patient's surgical wound were normal healing according to the Southampton scoring system without related complications (Figure 3).

## Discussion

The existing navigation system is a single-point display screen output mode, which cannot establish an effective spatial connection between the surgical planning image and the patient's actual anatomical structure. The surgeon needs to view the output of the tomographic image by the surgical planning and navigation system through the display screen and then imagine the actual relationship between the image x‘and the patient's anatomical structure. There is a problem with the spatial conversion. The doctor's repeated switching of the field of view between the operation area and the display screen will reduce the operating efficiency and affect the operation effect (6).

**Figure 4:**
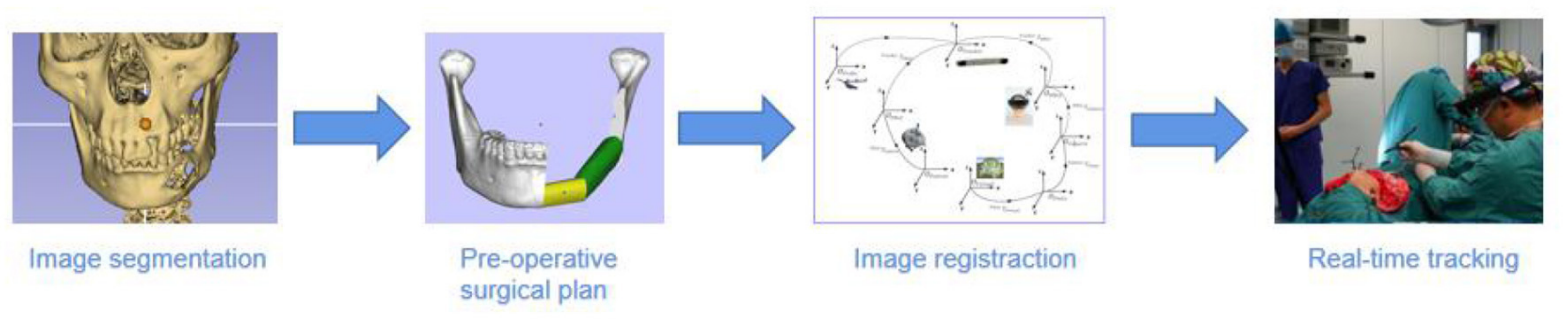
The main procedures of MR-based surgical navigation.

This paper introduces mixed reality technology into the existing navigation system, so the navigation system can output three-dimensional images and display them together with the patient's actual anatomy in real-time. Mixed reality equipment can assist the navigation system in integrating the surgical planning three-dimensional model with the actual patient's anatomy during the operation and complete the continuous spatiotemporal registration of the patient's surgical area anatomy and the planned three-dimensional model (8). The whole process includes importing the preoperative planning 3D model, the fusion registration of the 3D projection of the intraoperative planning model, and the patient's actual anatomical structure. And surgeon can see the patient's anatomical structure and the 3D model simultaneously in the operation field. Therefore, the introduction of mixed reality technology into the navigation system of oral and maxillofacial surgery can realize three-dimensional visual navigation, better solve spatial imagination and graphic conversion of existing navigation systems (10) (Figure 4).

**Figure 5:**
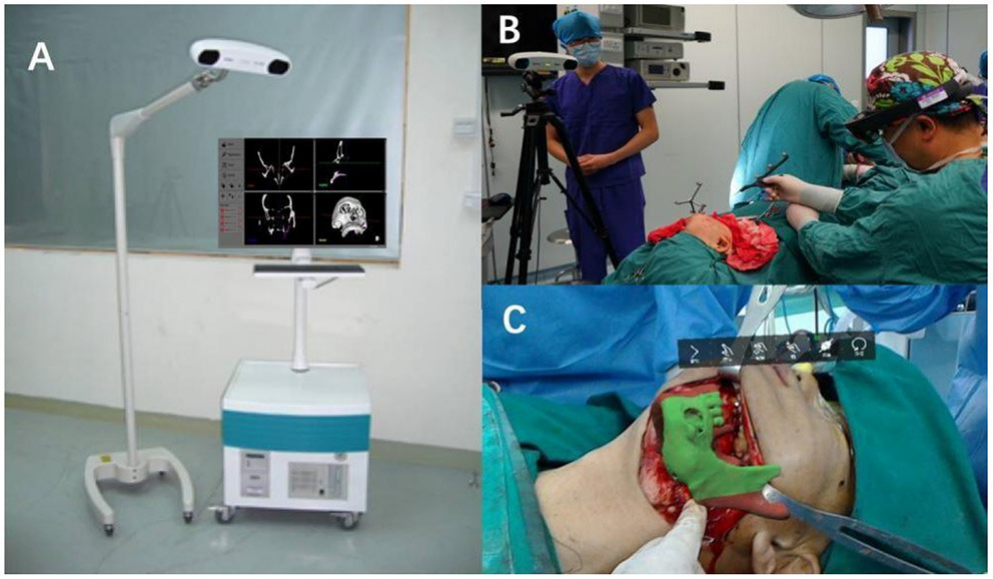
**(A)** The existing navigation system is a single-point display screen navigation. According to the tomographic image on the display screen, the surgeon needs to imagine the space independently and cannot observe the surgical field and the display screen simultaneously. **(B)** The working model of the mixed reality navigation system, which can directly display the surgical planning plan and three-dimensional virtual model in the surgical field, and the surgeon can adjust it through voice and gesture operations. **(C)** The head-mounted device uses the navigator to perform continuous intraoperative temporal and spatial configuration, directly realizing the superimposed display of the patient's anatomical structure and the virtual model in the surgical field.

The mixed reality display device used in this study is a commercial wireless headset (Hololens) developed by Microsoft Corporation in the United States. As the most advanced mixed reality display device, its display technology is optical transmission (near-eye 3D diffraction) display. Unlike the more researched video-based display technology, the visual transmissive display can integrate the three-dimensional model image and the natural ambient light in the translucent lens, thereby completely restoring the three-dimensional human vision (11). The three-dimensional model is directly superimposed on the patient's actual surgical area and maintains consistency with the patient's corresponding anatomical structure through continuous Spatio-temporal registration. Therefore, the surgeon can visually observe the patient's internal anatomy in the surgical field of view, accurately determine the resection range, and avoid secondary injuries. In addition, the device is operated through gesture recognition and voice control, avoiding physical contact during use, providing conditions for its application in surgery. The US Microsoft Corporation launched a commercial head-mounted mixed reality display device (Hololens) in 2015 and used it in mechanical processing and manufacturing. Due to its excellent three-dimensional display performance quickly gained clinical applications in the medical, especially the surgical field. Scholars such as Sun et al. (12) first reported using mixed reality technology to assist accurate breast tumor resection in a domestic journal in 2017; Tepper et al. (13) also said using mixed reality technology to help jaw reconstruction surgery in the same year. Based on the excellent optical performance of HoloLens and the open software development environment, more and more scholars have begun to use this device for mixed reality technology-assisted surgery-related research in the past 2 years (Figure 5).

In the research of Chinese scholars, mixed reality technology has been reported in neurosurgery, orthopedics, thoracic surgery and other fields for preoperative planning and doctor-patient communication. Scholar Yang Dezhen used mixed reality technology to holographically display individualized 3D virtual models of tumors, blood vessels, brain tissues, and others. They were used for preoperative planning, intraoperative guidance, preoperative talks, and teaching rounds. Research shows that mixed reality technology can display individualized holographic images of patients in a three-dimensional and intuitive manner in any scene. So that surgeons, patients and family members can have a comprehensive understanding of the lesion and surrounding anatomical structures, which improves surgeons' understanding of the anatomy of the operation area and the efficiency of doctor-patient communication. Huang et al. (14) Scholars use mixed reality technology to assist surgical treatment of osteoporotic femoral neck fractures. This technology can shorten the operation time while optimizing the femoral neck nail passage, increasing the femoral neck cortical support, and improving the efficacy; Ma et al. (15) Scholars have introduced mixed reality technology in the percutaneous kyphoplasty of the spine, which accurately displays the compression fracture site and surrounding anatomical structures in three dimensions during the operation. The operation path is clear, and the compression fracture forming site can be accurately located; Tang et al. (16) and Ma et al. (17) tried to introduce mixed reality technology in the field of thoracic surgery and achieved good results in the precise resection and reconstruction of chest wall tumors and the precise positioning and resection of small pulmonary nodules. Tang et al. (18) has completed preoperative three-dimensional reconstruction, intraoperative three-dimensional display and postoperative evaluation for eight patients with head and neck tumors. Although they successfully completed the operations with a control error of 5 mm, their system is mainly used for intraoperative display and lacks an effective positioning tracking system.

Mixed reality technology-assisted surgical navigation is also a hot topic in this field by international scholars in the past 2 years. In 2018, García-Vázquez and other scholars from the University of Lübeck in Germany (19) discussed the possibility of using mixed reality technology to assist endovascular aortic repair (EVAR) in the field of vascular surgery, and found that three-dimensional visual display can be a better alternative Intraoperative angiography reduces radiation exposure and the use of contrast agents, but its calculation speed and intraoperative registration accuracy cannot meet clinical needs; in 2019, Checcucci and other scholars from the University of Turin, Italy (20) performed nephron-preserving surgery in the urology department (NSS) tried to use mixed reality display equipment for three-dimensional visual surgery planning. Research shows that using mixed reality display equipment to assist surgery planning has better performance in plan display and anatomical accuracy, which helps surgeons better understand surgery Complexity, but the research is limited to preoperative planning and presentation and does not involve intraoperative navigation; in the field of Otorhinolaryngology-Head and Neck Surgery, American Rose and other scholars (21) affirmed the superiority of mixed reality display technology in a three-dimensional display. And its potential value in improving surgical efficiency and patient safety also pointed out the need to further enhance the computing speed and registration accuracy of head-mounted devices to meet clinical needs.

Worldwide scholars agree that mixed reality technology-assisted surgery has broad application prospects, and it is becoming a hot research topic in digital surgery. However, although the current mixed reality display device (Hololens) can scan the surrounding environment in real-time through a depth camera for positioning, it is limited by hardware conditions. Its positioning accuracy and speed cannot meet the needs of surgical operations. Pepe et al. (22) tried to improve the system's accuracy by improving the registration algorithm. The average error in the three-dimensional direction was reduced to 3–5 mm, but it still could not meet the needs of oral and maxillofacial surgery. Therefore, it is necessary to improve further the response speed of mixed reality equipment and the accuracy of intraoperative registration in the following research.

At this stage, the related research involving improving the registration accuracy of mixed reality devices is trying to improve the system response speed and intraoperative registration accuracy through the software development and algorithm optimization of the mixed reality display device (Microsoft's HoloLens) (22, 23). Scholars such as Faith (24) tried to use manual registration methods to improve the registration accuracy of mixed reality display devices. Studies have shown that the deviation from standard neurosurgical navigation systems reaches 4 mm, which still cannot meet clinical needs. At present, it is generally believed that the mixed reality display device (HoloLens) is an essential factor that limits its registration accuracy. Because the volume and weight of the head-mounted device are strictly limited, the accuracy of the depth camera and the computing power of the processor used is far inferior to that of the desktop Class equipment (25). The limitation of hardware restricts the improvement of the mixed reality system's overall registration accuracy and operation speed by this research idea. This paper innovatively proposes and realizes the research idea of combining mixed reality display devices with high-precision optical navigation systems.

By leveraging the advantages of the existing optical navigation system's computing power and positioning accuracy, the overall registration accuracy of the mixed reality navigation system is improved. And computing speed (10). Moreover, the maxillofacial surgery planning software and navigation system involved in this article are all independently developed by the project team, with critical technologies and systems with independent intellectual property rights, which have broad clinical transformation and application prospects (26–28). However, the system still needs some preliminary data preparation work. The overall registration speed and overall accuracy cannot fully meet the clinical needs, that is, the target average error for bone tissue is controlled at 1 mm. There is still room for improvement in real-time registration and tracking and the surgeon has certain equipment learning requirements.

## Conclusions

The maxillofacial digital surgery system based on mixed reality technology can superimpose and display three-dimensional navigation information in the doctor's surgical field of vision, which solves the problem of visual conversion and space conversion of the existing navigation system work efficiency of digitally assisted surgery. It improves the work efficiency of digitally assisted surgery, effectively reduces the surgeon's dependence on spatial experience and imagination, and protects important anatomical structures during surgery. It is a significant clinical application value and potential. However, the current cross-linking speed of the mixed reality display device and the navigation system and the system's overall accuracy still cannot fully meet the clinical needs. Further research is needed to improve the system accuracy and response speed.

## Data Availability Statement

The original contributions presented in the study are included in the article/supplementary material, further inquiries can be directed to the corresponding author/s.

## Ethics Statement

The studies involving human participants were reviewed and approved by Independent Ethics Committee of Shanghai Ninth People's Hospital Affiliated to Shanghai Jiao Tong University School of Medicine. The patients/participants provided their written informed consent to participate in this study. Written informed consent was obtained from the individual(s) for the publication of any potentially identifiable images or data included in this article.

## Author Contributions

TJ and XC designed the experiments. RY and CL collected the data. PT and AA did the calculation. RY wrote the main manuscript text and all authors approved the manuscript.

## Funding

Shanghai Jiaotong University Medical Engineering (Science) Cross Fund, Project Number: YG2019ZDA06 and Shanghai JiaoTong University School of Medicine, Shanghai Ninth People's Hospital Cross Fund Funded, Project Number: JYJC201807.

## Conflict of Interest

The authors declare that the research was conducted in the absence of any commercial or financial relationships that could be construed as a potential conflict of interest. The reviewer HY declared a shared affiliation with several of the authors RY, CL, PT, TJ, and XC to the handling editor at the time of the review.

## Publisher's Note

All claims expressed in this article are solely those of the authors and do not necessarily represent those of their affiliated organizations, or those of the publisher, the editors and the reviewers. Any product that may be evaluated in this article, or claim that may be made by its manufacturer, is not guaranteed or endorsed by the publisher.
